# Novel Homozygous Pathogenic Mutations of LAMA 2 Gene in Patients with Congen ital Muscular Dystrophy

**DOI:** 10.22037/ijcn.v15i1.21649

**Published:** 2021

**Authors:** Negar KHODAENIA, Zahra FARJAMI, Amir Hosein ASHNAEI, Neshat EBRAHIMI, Navid CHELVARFOROOSH, Andoni URTIZBEREA, Ehsan RAZMARA, Massoud HOUSHMAND

**Affiliations:** 1 National Institute of Genetic Engineering and Biotechnology, Tehran, Iran; 2 Department of Modern Sciences& Technologies, Medicine Faculty, Mashhad University of Medical Sciences, Mashhad, Iran; 3 Laboratory of Cedars-Sinai Medical Center, Los Angeles, California, USA; 4 Department of Agricultural Biotechnology, Science Faculty, Ferdowsi University of Mashhad, Mashhad, Iran; 5 Institut de Myologie, Hopital de la Salpetriere, Paris, France; 6 Department of Molecular Genetics, Faculty of Biological Science, Tarbiat Modares, Tehran, Iran

**Keywords:** LAMA2, DMC1A, Muscular Dystrophy

## Abstract

The laminin α2 subunit is a protein encoded by the laminin α2 gene(LAMA2) which has the role of adhesion (attachment of cells to one another). Genetics consideration showed that mutation in LAMA2 caused a collection of muscle-wasting conditions called muscular dystrophy. This disorder causes disconnection of muscular cells and degeneration of the musculoskeletal system. In this study, we defined the molecular consideration of three patients with laminin α2 deficiency by clinical presentations of congenital muscular dystrophy. In this regard, 65 exons of the LAMA2 gene were amplified by polymerase chain reaction. Moreover, multiple ligation-dependent probe amplification and next generation sequencing (NGS) were carried out for all the patients. Because of NGS negativity, gene sequencing was performed. Results of searching for rearrangements of the LAMA2 gene enabled us to recognize homozygous pathogenic mutations c.2049_c.2050del, c.7156-2A>G, and c,1303C>T. These mutations produce an out-of-frame transcript that will be degraded by nonsense mediated decay. Therefore, we think these changes are pathogenic ones.

## Introduction

The laminin α2 (LAMA2) gene contains subunits of a certain protein family called laminins. Laminins are heterotrimer proteins made of α, β, and γ subtypes that have distinct tissue-specific expression patterns (1). They are thought to mediate migration, attachment, and organization of cells into tissues during fetal development by interacting with other extracellular matrix molecules (2).  LAMA2is located in 6q22-q23 which encodes the alpha 2 chain (one of the subtypes of laminin 2 (merosin) and laminin 4 (s-merosin)). Mutations in the LAMA2 chain gene (LAMA2 gene) caused congenital muscular dystrophy (CMD). This disease is a group of hereditary muscle disabilities causing increased weakening and breakdown of skeletal muscles (3). Children with this condition have normal intelligence and, in some cases, seizures (4). Histological studies showed that large variations occurred in muscle fiber size, a few regenerating and necrotic of fibers. Diagnosis is based on clinical characteristics and morphological changes (5). The DMC1 protein is a meiosis-specific DNA recombinase between homologous chromosomes. Two types of the DMC1 protein have been characterized and include DMC1A and DMC1B (6). Genetic recombination in meiosis has a significant role in making diversity in genetic information and enables reduced segregation of chromosomes that are necessary for creation of gametes throughout sexual reproduction (7). There is no obvious procedure that explains muscle fiber degeneration in CMD (8). Numerous duplications and deletions in some exons of LAMA2 are related to CMD (9). This study was conducted to assess novel homozygous pathogenic mutations in patients with CMD phenotype.

## Material &Methods

**Patients**

Case 1 was 10 years old (the exact time of birth was not provided) and presented with merosin deficiency. The patient was originally from Kuwait. Case 2 was a child who was born on August 25, 2016, and presented with a merosin deficiency. Case 3 was a child (birth date was not available) deceased because of DMC1A. The next generation sequencing (NGS) analysis was negative for all the patients, and thus, gene sequencing was performed.

**DNA Extraction:** Genomic DNA was extracted from the patients’ blood samples and isolated using DNA Extraction Kit YTA (Cat No. YT 9040) in accordance with manufacturer's instructions (10, 11). 

**PCR:** DNA samples of all the three patients were investigated by PCR amplification on 65 exons of the LAMA2 gene as well as on intronic boundaries and analyzed with Sequencer and Sequencing Analysis Software. For PCR, we used a 20 μL sample containing 1 μL Forward Primer, 1 μL Reverse Primer, 6 μL Diluents Water, 2 μL DNA 50 ng/ml, and 10 μL Master Mix.

**DNA Sequencing:** Sequencing of PCR products was confirmed by Sanger sequencing (12). Deletion/duplication analysis was carried out by multiple ligation-dependent probe amplification (MLPA-P391-A1 and P392-A1, LAMA2).

## Results

The search for small rearrangements of the LAMA2 gene allowed us to identify a homozygous pathogenic mutation c.2049_c.2050del (according to the international HGVS nomenclature applied to the transcript NM_000426.3) in the DNA of Case 1, a homozygous path genetic mutation c.7156-2A>G in Case 2, and a homozygous pathogenic mutation c,1303C>T in Case 3.

**Table 1:** Summary of DNA sequencing results

Patients	Exon12	Exon12
p1	c.7938 G>A hetero p.Met 2649 Ile	-
p2	-	c.1798G>A homo p.G600R
p3	c.7938 G>A hetero p.Met 2649 Ile	-
p4	-	-
p5	c.7938 G>A hetero p.Met 2649 Ile	-
p6	-	-
p7	-	-
p8	c.7938 G>A hetero p.Met 2649 Ile A	-
p9	-	-
p10	hetro c.8076+6G>A	-
p11	c.7938 G>A hetero p.Met 2649 Ile	-
p12	c.7938 G>A hetero p.Met 2649 Ile	c.1856G>A homo p.R619 H bening polymorphism
p13	c.7938 G>A hetero p.Met 2649 Ile	-
p14	c.7938 G>A hetero p.Met 2649 Ile	-
p15	c.7938 G>A hetero p.Met 2649 Ile	-
p16	-	c.1856 G>A homo p.R619 H polymorphism
p17	-	-
p18	-	-
p19	-	-
p20	-	-

## Discussion

The LAMA2 chain contains 64 exons and is found in a complicated lattice of proteins and other molecules that forms in spaces between cells (the extracellular matrix). Here, laminins help control adhesion of cells to one another (13). It is still a challenge to obtain an exact molecular diagnosis with present genetic methods due to the genetic heterogeneity and clinical of this condition (14). Muscular biopsy appears to be an essential procedure to confirm the CMD condition. However, the current investigation proposed that molecular genetic analysis may be a better way compared to muscular biopsy if clinical phenotypes confirm CMD diagnosis (15, 16). We described herein three patients with rare mutations of the LAMA2 gene (c.2049_c.2050del, c.7156-2A>G, and c,1303C>T homozygous pathogenic mutations). Case 1 was diagnosed with CMD, and NGS was performed for him. However, the results did not reveal his condition, and thus, gene sequencing was performed. Afterward, the c.2049_c.2050del homozygous pathogenic mutation was observed. This variation is not detected in large population cohorts (17, 18). In Case 2, NGS was carried out, but the results did not show the mutation; therefore, gene sequencing was performed. The c.7156-2A>G mutation inhibited the receptor of the exon 51 and most probably induced merosin absence. In Case 3, gene sequencing was performed due to NGS negativity. The c,1303C>T homozygous pathogenic mutation was observed. The case deceased because of DMC1A after birth. The case’s parents appeared to be healthy carriers, and thus, their son could be homozygous for this mutation.

## In Conclusion

Cases 1 and 2 presented with the c.2049_c.2050del and c.7156-2A>G homozygous pathogenic mutations of the LAMA2 gene, respectively. Deletion of the exon 14 caused c.2049_c.2050 mutation, and c.7156-2A>G mutation abolished the acceptor splice site of the exon 51. These mutations produce out-of-frame transcripts that will be degraded by nonsense mediated decay. Therefore, the patients presented with DMC1A. These homozygous pathogenic mutations were probably inherited from the both parents. Case 3 presented with the c,1303C>T (p. Arg435*) pathogenic mutation of the LAMA2 gene. The case’s parents were healthy heterozygous carriers of this pathogenic mutation, and thus, their son was probably homozygous for this mutation. The risk of repeating this condition was estimated to be 25% for future pregnancies of this couple. Since these mutations are inherited and can occur in the next generation, we can perform a prenatal diagnosis in these families if necessary and/or study additional family members for genetic counseling.
